# Experiences, attitudes, and knowledge of medical students regarding intellectual and developmental disability: a Canadian study

**DOI:** 10.1186/s12909-024-06482-z

**Published:** 2024-12-20

**Authors:** Gabriel Tarzi, Alyssa Yip, Muhammad Irfan Jiwa, Anupam Thakur, Soumya Mishra, Laura Koch, Olivia Mendoza, Amandi Perera, Lachina Mckenzie, Yona Lunsky

**Affiliations:** 1 Azrieli Adult Neurodevelopmental Centre, Centre for Addiction and Mental Health, 1001 Queen St W, Toronto, ON M6J 1H4 Canada; 2 Department of Psychiatry, University of Toronto, Toronto, ON Canada

**Keywords:** Intellectual disability, developmental disability, medical education, clinical curriculum, medical students

## Abstract

**Background:**

As the healthcare of individuals with intellectual and developmental disabilities (IDD) shifts toward community-based services, physicians in all areas of medicine are more likely to care for this population. To ensure that all physicians can provide high-quality care to people with IDD, further understanding and attention to undergraduate medical education related to IDD is needed.

**Methods:**

A 24-item survey assessed the experiences, attitudes, knowledge, skills, and future interest of Canadian medical students regarding IDD. Descriptive statistics were calculated for questionnaire responses and responses of students who had more in-depth experience were compared to those of students with minimal past experience.

**Results:**

A total of 443 Canadian medical students completed the survey. Students did not feel competent obtaining clear histories from people with IDD. Most students were not confident they could provide quality care to this population but wanted further learning. Students with prior IDD experiences through family/friends felt more knowledgeable and interested in caring for this population than those with community/clinical and minimal experiences.

**Discussion:**

Many Canadian medical students lack the knowledge and skills needed to adequately care for people with IDD. Despite this, a majority of students were interested in further learning opportunities to improve care for people with IDD. These findings underscore the necessity of evaluating the current medical curriculum and implementing measures to better prepare students to care for this population.

## Background

People with intellectual and developmental disabilities (IDD) can have complex health needs which are poorly addressed in Canada and around the world [1, 2]. Adults with IDD face health disparities due to difficulties in accessing healthcare, inadequately trained health care workers, and lack of access to preventative and management medicine [3]. As the healthcare management of people with IDD shifts away from institutions towards community-based services, adequate access and training is becoming more important than ever before, as physicians in every area of medicine are more likely to care for people with IDD [4].

Unfortunately, studies have shown that some healthcare staff lack confidence in their ability to meet the health needs of people with IDD and hold negative attitudes towards caring for this population [5, 6]. Patients and families report that these difficulties in accessing high quality health care for people with IDD are problematic and compound with existing systems-related disparities leading to negative health outcomes [7, 8]. Some countries, such as the United Kingdom, have begun mandating training for all health care professionals to help address these issues. However, having provisions for training while in school can also shape attitudes and inform about roles and responsibilities as they are being formed [9, 10]. In addition, research on medical training in IDD has highlighted that increased education and training is an effective avenue to increasing confidence in providing clinical care for people with IDD [11, 12]. Therefore, it is imperative that medical students, as future physicians, be adequately trained in providing clinical care for people with IDD to shape positive attitudes and increase confidence in addressing the health needs for this population.

Much of the research related to IDD and healthcare training pertains to medical residents and practicing healthcare professionals; there is limited research on undergraduate medical education. International research on pre-graduate/undergraduate medical education has demonstrated that didactic and clinical interventions increase medical students’ knowledge, understanding, and confidence surrounding care of individuals with IDD [13, 14]. Medical students’ attitudes towards caring for individuals with IDD have also been shown to improve with increased clinical experiences working with this population [15], although there can be risks when teaching in clinical settings where problems are often medicalized or the population is stigmatized [16]. It has been suggested that increasing students’ experiences with people with IDD when they are well and outside of healthcare settings, particularly outside of hospitals, may increase willingness to care for this population [14, 17, 18]. However, in countries with data on IDD medical education, there has been little progress in recent years to address medical students’ knowledge gaps and increase positive attitudes towards caring for this population [14, 19–21]. In addition to increasing medical students’ knowledge, it is pertinent to investigate their attitudes towards working with this population and the factors that influence their attitudes. As attitudes change over time, it is important to develop curricula based on students’ current experiences, attitudes, interests, and needs.

An earlier survey of senior medical students at two Canadian medical schools demonstrated overwhelming support for curriculum changes with an emphasis on increased contact with people with IDD in clinical settings [22]. Two more recent studies of pre-clerkship medical students at the University of Toronto found that despite students being knowledgeable about IDD, they did not feel competent in their clinical skills when caring for this population [16, 23]. Canadian undergraduate medical students’ desire for further training is consistent with international research that emphasizes the value of sufficient medical training in caring for people with IDD [13, 14, 24, 25]. There is limited literature on what medical students are being taught regarding the clinical care for people with IDD, but as evident by practitioners’ gap in communication skills and general knowledge of IDD, there is a clear need for further clinical teaching regarding IDD [5].

The aims of this study were to further understand the experiences, attitudes, knowledge, clinical skills, and future interests of Canadian undergraduate medical students in working with people with IDD and the association between previous experiences to IDD on these measures. Further understanding of medical students’ needs and gaps in knowledge can inform medical educators on interventions and clinical training that can be implemented as an important step to promoting equitable care for all.

## Methods

A 24-item survey reporting on the experiences, attitudes, knowledge, self-perceived clinical skills, and interests towards IDD was distributed to Canadian undergraduate medical students. It was circulated through the various medical student societies at the national and provincial level, as well as through medical school student associations/councils, social media, and word of mouth. Eleven of seventeen school medical societies or registrar’s offices agreed to circulate information about the study to all of their students. Participants were eligible to participate in this study if (1) they were an undergraduate medical student currently attending a Canadian school and (2) were able to provide consent to participate in this study. The survey was distributed between November 2021 and August 2022. Survey responses were anonymously collected through REDCap and were approved by the Centre for Addiction and Mental Health Research Ethics Board (090/2021).

### Survey Items

The survey was an adaptation of the questionnaire used by Jiwa et al. (2020) [23]. It also included questions pertaining to subjective knowledge and skills related to diagnosis and management of IDD [26], attitudes [27], and items used in surveys described by Werner and Stawsky (2012) [28]. Measures and constructs described in these studies have been utilized in other studies investigating knowledge, attitudes, and perspectives of health professionals regarding IDD.

The survey was subdivided into five sections: prior experiences, attitudes, knowledge and skills, future interests, and student demographics. In the experience section, participants reported the types of prior experiences they have had with people with IDD, including outside healthcare settings. Attitudes were assessed through 7 items on their comfort, confidence, and beliefs surrounding caring for someone with IDD. The knowledge and clinical skills section consisted of 12 items related to different aspects of care for individuals with IDD. Students were also asked to what extent they anticipated and were interested in caring for people with IDD in their future practice. Lastly, demographic data related to the participants were collected. Most questions were five-point Likert-type responses from “Strongly Agree” to “Strongly Disagree”. Other response types include true or false, open response, multiple-selection, or continuous scale ranking from 0 to 10.

### Data analysis

Descriptive statistics were calculated for each section of the survey to explore experiences, knowledge, attitudes, and future interests caring for people with IDD and training in IDD. Descriptive statistics include means and standard deviation for continuous variables and ratios and frequencies for categorical variables.

The responses from the attitude, knowledge and clinical skills, and future interests sections were further analyzed based on the participants’ prior experiences with IDD. Participants were assigned to one of three groups based on their reported prior experiences. Participants who indicated they had experience with IDD through immediate family member(s), extended family members(s), or family friend(s) were assigned to the “Family/Friends” group. If respondents indicated they had experience with IDD through school peer(s), employment-related, clinical placement/clinical experience, or volunteering, but not through family or friends, they were assigned to the “Community/Clinical” experience group. Participants who indicated that they had minimal/none, post-secondary coursework, or experience through a research project, and did not have any family/friends or community experience, were assigned to the “Minimal/None” experience group.

Comparisons between groups were conducted using ANOVA, χ^2^ test, or Fisher’s exact test, as appropriate. A significance level of *p* = 0.05 was used for the analysis. Participants with greater than 30% missing data were excluded from analysis; other missing data were handled using pairwise deletion.

## Results

A total of 467 people consented to participate in the study. After accounting for missing data and inclusion criteria, 443 undergraduate medical students from across 16 different medical schools in Canada were included. Over half of respondents (*n* = 244) were between 20 and 25 years of age and the majority identified as women (*n* = 253). The most represented school was the University of Toronto with 129 participants. Just over half of participants (*n* = 230) were in either their first or second year of study at the time of completion of the survey.

### Experiences

A total of 443 participants completed this portion of the survey. Over a quarter of respondents indicated they had “minimal/none” experience (*n* = 125, 28.2%), and of these 125 respondents, 86 selected only “minimal/none” and did not select any other experience. The most common prior experience was volunteering, endorsed by 187 (42.2%) of the respondents. Other experiences included immediate family (*n* = 51, 11.5%), extended family or family friends (*n* = 126, 28.4%), school peers (m = 121, 27.3%), employment (*n* = 106, 23.9%), clinical placement/experience (*n* = 99, 22.3%), post-secondary coursework (*n* = 68, 15.3%), and research projects (*n* = 29, 6.5%). Of respondents that selected “other” experiences, responses included “self” (*n* = 4), neighbours (*n* = 2), friends (*n* = 1), and shadowing (*n* = 1). Less than a third of participants received any IDD education as part of their training, and this only increased slightly (36%, *n* = 154) when considering only senior medical students, defined as third- and fourth-year students completing clinical rotations.

When grouped by experience responses, 156 (35.2%) of respondents were in the Family/Friends experience group, 185 (41.8%) of respondents were in the Community/Clinical experience group, and 96 (21.7%) of respondents were in the Minimal/None experience group. Participants that selected “other” as their only experience (*n* = 6) were not assigned a group because it was not obviously apparent where best to categorize them.

### Attitudes

In total, 406 participants completed the portion of the survey pertaining to their attitudes about people with IDD. Most students reported feeling moderately comfortable interacting with someone with intellectual and developmental disability; the average ranking of confidence on a scale of 0–10 was 6.5 ± 2.3. However, students on average did not report feeling competent in obtaining a clear history from someone with IDD, with an average response of 4.5 ± 2.3. When asked whether students felt confident communicating with someone with IDD, the average ranking of confidence was 5.9 ± 2.3. Responses for all three questions varied greatly, with ranges of 0–10.

When asked about their beliefs around virtual care for people with IDD, 86.2% (*n* = 350) of respondents indicated that healthcare professionals need more training in using virtual care for people with IDD. When asked about the value of virtual care for caregivers of people with IDD, 77.3% (*n* = 314) of respondents agreed or strongly agreed with its value.

When stratifying the attitude responses based on the participants’ experience group, two respondents did not complete both sections and were excluded from analysis (*n* = 404) (Table 1). Confidence in communication with someone with IDD significantly differed between groups (F(2,401) = [4.94], *p* < 0.05) and was greatest in the Family/Friends experience group. Comfort interacting and competence in obtaining a clear history from someone with IDD did not statistically differ between groups. In terms of agreement with the statements provided in Table 1, there were no statistical differences between the Family/Friends, Community/Clinical, or Minimal/None experience groups. It is clear that across all experience groups there is a desire to improve care for individuals with IDD with nearly two thirds of each group indicating they would like further resources and learning opportunities.

**Table 1:** Participants’ attitudes towards health care of individuals with intellectual and developmental disabilities, stratified by experience groups

Item	Total (*n* = 404)	Family/ Friends (*n* = 139)	Community/Clinical (*n* = 176)	Minimal/ None (*n* = 89)	*p*-value
**On a scale from 0–10, to what extent do you feel…**	**Mean ± SD**				
Comfortable interacting with someone with an IDD?	6.5 ± 2.3	6.9 ± 2.2	6.5 ± 2.2	6.0 ± 2.4	F(2,401) = [2.03], *p* = 0.1325
Confident communicating with someone with an IDD?	5.9 ± 2.3	6.2 ± 2.2	5.9 ± 2.3	5.3 ± 2.3	F(2,401) = [4.94], *p* = 0.008
Competent in obtaining a clear history from someone with an intellectual and developmental disability?	4.5 ± 2.3	4.6 ± 2.2	4.6 ± 2.4	4.0 ± 2.3	F(2,401) = [2.09], *p* = 0.1248
	**% agree or strongly agree**				
Healthcare professionals need more training in using virtual care for people with IDD.	86.5	89.2	86.4	83.2	*p* = 0.419
Virtual care is very valuable for caregivers of people with IDD.	78.5	77.0	82.3	69.7	*p* = 0.061

### Knowledge and perceived clinical skills

A total of 404 participants completed the knowledge portion of the survey. When caring for adults with IDD, 42.8% (*n* = 173) of participants felt that they were skilled in adapting communication and clinical approach for each patient. Common adaptations to communication identified by participants included talking slower, using simplified language, using the patient’s preferred communication style, and checking the patient’s understanding frequently throughout the conversation. A total of 154 participants (38.1%) reported feeling confident that they can provide quality care for adults with IDD. However, only 23.5% (*n* = 95) reported being knowledgeable about common comorbidities and care issues that affect this population and 21.4% (*n* = 86) reported that they were familiar with community resources for this group. When compared to communicating with caregivers, 58.4% (*n* = 236) of participants reported feeling confident discussing the individual’s disability with a caregiver, while only 38.6% (*n* = 156) reported confidence in discussing this with the patient themselves.

The majority of participants, 87.1% (*n* = 352), knew that a diagnosis of IDD is based on more than just intelligence testing. Likewise, 87.4% (*n* = 353) recognized that people with IDD tend to have more complex/overlapping health needs. However, 30.2% (*n* = 122) mistakenly believed that most adults with IDD live in a group home setting.

Responses from the knowledge portion of the survey were stratified based on experience groups (Table 2). The knowledge and confidence in providing care to individuals with IDD differed between experience groups, with prior experience resulting in participants feeling more prepared and skilled. The Family/Friends experience group felt more comfortable discussing a patient’s disability with the patient (*p* < 0.05) and the caregiver (*p* < 0.05) compared to the Community/Clinical and Minimal/None experience groups. The Family/Friends experience group also felt more knowledgeable about common comorbidities (*p* < 0.05), community resources (*p* < 0.05), and increased confidence in the care they can provide (*p* < 0.05), in assessing behavioural challenges (*p* < 0.05), and adapting communication skills (*p* < 0.05) than the Community and Minimal/None experience groups. The Community/Clinical experience group was more confident in these skills than the Minimal/None experience group. Compared to the Family/Friends and Community/Clinical experience groups, the Minimal/None experience group was more likely to believe that most adults with IDD live in a group home setting (*p* < 0.05).

**Table 2:** Participants’ perceived clinical skills and knowledge regarding intellectual and developmental disability health care, stratified by experience groups

Variable	Total (*n* = 404)	Family/ Friends (*n* = 139)	Community/Clinical (*n* = 176)	Minimal/ None (*n* = 89)	*p*-value
	**% that answered Agree or Strongly Agree**				
Confident in my ability to clinically recognize that he/she has IDD	29.5	35.2	33.5	12.4	*p* = 0.0003
Comfortable in discussing the individual’s disability with the patient	38.6	54.7	35.2	20.2	*p* < 0.00001
Comfortable in discussing the individual’s disability with the caregiver	58.4	71.2	54.0	47.2	*p* = 0.0004
Confident that I can provide quality care to the individual	38.1	50.4	36.9	21.4	*p* = 0.00006
Confident in assessing behavioural challenges	24.3	31.6	25.6	10.1	*p* = 0.00091
Knowledgeable about common co-morbidities and care issues affecting patients with IDD (including abuse)	23.5	31.6	22.7	12.4	*p* = 0.0034
Familiar with other community resources for people with IDD	21.4	26.6	23.3	7.9	*p* = 0.0020
Skilled in adapting my communication and approach to the patient’s needs	42.8	55.4	43.2	22.5	*p* < 0.00001
**True/False Statements**	**% that answered correctly**				
Most adults with IDD live in a group home setting.	69.8	70.5	74.4	59.5	*p* = 0.043
An IDD diagnosis is strictly based on IQ.	87.1	87.1	86.9	87.6	*p* = 0.9863
People with IDD tend to have more complex/overlapping health needs.	87.4	87.1	87.5	87.6	*p* = 0.9893
People with IDD always need substitute decision makers to make decisions about their health care.	85.4	85.6	84.1	87.6	*p* = 0.7389

### Medical training in IDD and future interests

Of the survey respondents, 26% (*n* = 100) reported they expected to provide significant care (“quite a bit” or “a lot”) to people with IDD in their future practice. Similarly, 30% (*n* = 116) expressed strong interest (“quite a bit” or “a lot”) in providing care to people with IDD. Over half of participants (63.8%; *n* = 259) indicated that improving care for people with IDD is important and that they would like to be connected with more resources and learning opportunities, while 24.1% (*n* = 98) indicated that while they agree it is important, they may not have the time or resources to commit to further training. Eight percent of participants (*n* = 33) indicated that they were already involved in activities to support individuals with IDD, and only 3.0% (*n* = 12) of participants were not interested in learning more about IDD. There were four non-responders to this question.

When stratifying the responses on future interests based on experience group (Table 3), the Family/friends experience group was more likely to anticipate caring for people with IDD than the Community/Clinical and Minimal/none experience groups (*p* < 0.05). This same pattern was also true for interest in caring for individuals with IDD in their future practice (*p* < 0.05). The Family/friends group was more likely to seek out elective education compared to Community/Clinical and Minimal/none groups (*p* = 0.041).

**Table 3:** Participants’ future interest in caring for people with intellectual and developmental disabilities, stratified by experience groups

Variable	Total	Family/ Friends	Community/Clinical		Minimal/ None	*p*-value		
	**% “Quite a bit” or “A lot”**							
To what extent do you **anticipate** caring for people with intellectual and developmental disabilities in your future practice?	26% (*n* = 386)	35% (*n* = 136)	25% (*n* = 169)		12% (*n* = 81)	*p* < 0.001		
To what extent are you **interested** in caring for people with intellectual and developmental disabilities in your future practice?	30% (*n* = 384)	44% (*n* = 135)	26% (*n* = 168)		15% (*n* = 81)	*p* < 0.001		
		% Agree /	Strongly Agree					
I am not interested in learning more about IDD.	3.0 (*n* = 404)	2.2 (*n* = 139)	4.0 (*n* = 176)		2.2 (*n* = 89)	*p* = 0.577		
Improving care for people with IDD is important, but I’m not sure I have the time or resources to commit to further training.	24.2 (*n* = 404)	20.9 (*n* = 139)	27.0 (*n* = 176)		23.6 (*n* = 89)	*p* = 0.414		
Improving care for people with IDD is important, and I would like to be connected with more resources and learning opportunities.	64.4 (*n* = 404)	65.5 (*n* = 139)	62.6 (*n* = 176)		65.2 (*n* = 89)	*p* = 0.838		
Improving care for people with IDD is important, and I am already involved in activities to support individuals with IDD, in healthcare or otherwise.	8.4 (*n* = 404)	11.5 (*n* = 139)	6.3 (*n* = 176)		7.9 (*n* = 89)	*p* = 0.242		
	**% “Yes”**							
Have you attended any elective education on IDD?	7% (*n* = 384)	10% (*n* = 135)	7% (*n* = 168)		1% (*n* = 81)	0.041		
Senior medical students	10% (*n* = 154)	12% (*n* = 51)	11% (*n* = 74)		3% (*n* = 29)			

## Discussion

This study investigated the experiences, attitudes, knowledge, clinical skills, and future interests of Canadian undergraduate medical students with respect to caring for people with IDD. Experience with IDD was most common through volunteer experiences followed by extended family member(s) or family friend(s), and school peers. Just over a quarter of respondents indicated they had “minimal/none” experience with IDD. The majority of respondents also felt moderately comfortable interacting and communicating with someone with IDD; however, they did not feel comfortable obtaining a clear history. The importance of providing care was recognized, with most students interested in learning more about IDD and including this population in their future practice. A quarter of respondents, however, thought that while it is important, they did not have the time or resources to commit to improving care for people with IDD. Many respondents thought they could adapt their clinical communication skills to care for people with IDD, but very few felt knowledgeable about common care issues and community resources. Previous experience was associated with respondents feeling more comfortable, knowledgeable, and interested in caring for people with IDD.

These findings highlight the need for medical students to have increased teaching and clinical exposure to people with IDD during their undergraduate medical education. Students, particularly those with limited community or family experience prior, can have knowledge gaps which can influence care. For example, in this study the group with the least experience was more likely to be misinformed about where adults with IDD tend to live. Not being aware that adults with IDD tend to live independently or with family means that as future doctors, they are less focused on how to help people who rely on informal supports, or that they are less involved in future planning. Simply placing students in clinical settings without demonstrating how adults with IDD can live well in their communities can build clinical skills but it may also lead to sheltering attitudes [16]. For example, the distributions of experience and interest in caring for people with IDD show that students with Family/friends experiences are most interested in caring for this population. This increased willingness to care for people with IDD may be due to increased comfort interacting with people with IDD outside of health care settings [29]. Increasing early experiences to IDD outside of healthcare settings is therefore as important as providing learning opportunities within clinical care; otherwise, misconceptions about IDD that result in ableist attitudes can impact care - ignoring health issues and misattributing them to a person’s disability and denying treatment (diagnostic overshadowing) [30]. An educational intervention for medical students at the University of Toronto where students virtually toured IDD clinical settings without spending time with individuals in their community roles increased students’ perceptions that people with IDD need protection and supervision in daily activities [16]. Therefore, it is important that medical curricula balance clinical exposure with learning opportunities beyond the clinical settings to counter ableist perspectives.

Despite many medical students being interested in working with people with IDD in the future, students reported limited training experiences and feeling ill-equipped in certain areas. Individuals with IDD have increased rates of morbidity and mortality as a direct result of the healthcare system’s shortfalls [3, 7]. Learning from other jurisdictions, as well as from research with residents in psychiatry, family medicine, and pediatrics, involving people with lived experience of disability and family caregivers in education efforts is essential [31]. International research on IDD medical training has shown that when people with IDD are included in teaching medical students, students have increased comfort interacting and communicating with people with IDD [32–34, 36]. This has been implemented in certain Australian and Canadian medical curricula with positive effects on students’ self-reported competencies [14, 35]. Not every clinical educator will be an expert on caring for individuals with IDD, so it is pertinent that clinical educators utilize the expertise that individuals with lived experience can provide. Medical students appreciate the fact they need further training, particularly with clinical skills and virtual care for this population, but many do not seek out resources on their own. Although medical school curricula must cover a wide range of topics and skills, medical schools have a responsibility adapt their curriculum to satisfy these needs and to share courses and opportunities that work towards health equity for people with IDD.

### Limitations

One limitation of our study is the potential for selection bias with those with an interest in IDD being more likely to complete the study. Despite this survey being distributed through diverse mediums, many eligible students did not participate, and no additional information was provided to rule out a selection bias. Despite responses from nearly every medical school in the country (16 of 17), only nine surveys were completed in French and therefore do not adequately represent the perspectives of French speaking medical students. This survey did not provide a definition of IDD to participants and as such there may be variability in interpretation of survey items, impacting responses. Finally, this was a brief survey and does not allow nuances within teaching and experiences which can be done through interviews and more in-depth discussion.

## Conclusions

This study demonstrates that many Canadian undergraduate medical students lack the confidence, knowledge, and clinical skills to care for people with IDD. Prior experience seems to be important in influencing knowledge and interest in caring for people with IDD. Students were overwhelmingly supportive of improving care for people with IDD and the majority were interested in further learning opportunities. These findings highlight the need to review current medical curriculum surrounding IDD in Canada so that educators can highlight models of best practices and commit to expanding opportunities as part of broader equity and inclusivity efforts in various jurisdictions. As schools around the world embrace equity, diversity, and inclusivity efforts, it is important to know what students are experiencing across jurisdictions, and to share successes and innovations to improve care to this population.

## Data Availability

The datasets used and/or analysed during the current study available from the corresponding author on reasonable request.

## References

[1] Salvador-Carulla L, Saxena S. Intellectual disability: between disability and clinical nosology. Lancet. 2009;374(9704):1798–9. 19944847 10.1016/S0140-6736(09)62034-1

[2] Lunsky Y, Balogh R, Durbin A, Selick A, Volpe T, Lin E. The mental health of adults with developmental disabilities in Ontario: Lessons from administrative health data. Healthc Q. 2018;21(1):6–9. 10.12927/hcq.2018.25521 30051808

[3] Anderson LL, Humphries K, McDermott S, Marks B, Sisirak J, Larson S. The state of the science of health and wellness for adults with intellectual and developmental disabilities. Intellect Dev Disabil. 2013;51(5):385–98. 24303825 10.1352/1934-9556-51.5.385 PMC4677669

[4] Brown I, Radford JP. The Growth and Decline of Institutions for People with Developmental Disabilities in Ontario: 1876–2009. J Dev Disabil. 2015;21(2).

[5] Hemm C, Dagnan D, Meyer TD. Identifying training needs for mainstream healthcare professionals, to prepare them for working with individuals with intellectual disabilities: a systematic review. J Appl Res Intellect Disabil. 2015;28(2):98–110. 25266406 10.1111/jar.12117

[6] Iezzoni LI, Rao SR, Ressalam J, Bolcic-Jankovic D, Agaronnik ND, Donelan K, Lagu T, Campbell EG. Physicians’ Perceptions Of People With Disability And Their Health Care: Study reports the results of a survey of physicians’ perceptions of people with disability. Health Aff. 2021;40(2):297–306. 10.1377/hlthaff.2020.01452 PMC8722582 33523739

[7] Lin E, Balogh RS, Durbin A, Holder L, Gupta N, Volpe T, Isaacs BJ, Weiss JA, Lunsky Y. Addressing gaps in the health care services used by adults with developmental disabilities in Ontario. Toronto, ON, Canada: Institute for Clinical Evaluative Sciences; 2019.

[8] Shady K, Phillips S, Newman S. Barriers and facilitators to healthcare access in adults with intellectual and developmental disorders and communication difficulties: an integrative review. Rev J Autism Dev Disorders. 2022;30:1–3. 10.1007/s40489-022-00324-8 PMC9148936 35669718

[9] Jones J, McQueen M, Lowe S, Minnes P, Rischke A. Interprofessional Education in C anada: Addressing Knowledge, Skills, and Attitudes Concerning Intellectual Disability for Future Healthcare Professionals. J Policy Pract Intellect Disabil. 2015;12(3):172–80.

[10] Akbulut Zencirci S, Metintas S, Kosger F, Melekoglu M. Impact of a mixed method training programme on attitudes of future doctors toward intellectual disability. Int J Dev Disabil. 2022;31:1–7. 10.1080/20473869.2022.2085023 PMC10930146 38481456

[11] Adirim Z, Sockalingam S, Thakur A. Post-graduate medical training in intellectual and developmental disabilities: a systematic review. Acad Psychiatry. 2021;45:371–81. 33433827 10.1007/s40596-020-01378-8

[12] Ouellette-Kuntz H, Minnes P, Garcin N, Martin C, Suzanne Lewis ME, Holden JJ. Addressing health disparities through promoting equity for individuals with intellectual disability. Can J Public Health. 2005;96:S8–22. 10.1007/BF03403699 PMC6976115 16078552

[13] Vi L, Jiwa MI, Lunsky Y, Thakur A. A systematic review of intellectual and developmental disability curriculum in international pre-graduate health professional education. BMC Med Educ. 2023;23(1):329. 37170246 10.1186/s12909-023-04259-4 PMC10176941

[14] Clarke L, Tabor HK. The impact of inclusion: Improving medical student confidence in caring for adults with intellectual disabilities through an interactive, narrative-based session. J Intellect Dev Disabil 2023;7:1–5. 10.3109/13668250.2023.2198345 39815924

[15] Ryan TA, Scior K. Medical students’ attitudes towards people with intellectual disabilities: A literature review. Res Dev Disabil. 2014;35(10):2316–28. 24952372 10.1016/j.ridd.2014.05.019

[16] Berger I, Weissman S, Raheel H, Bagga A, Wright R, Leung F, Loh A, Lee-Jones C, Isaacs B, Vogt J. Evaluating the impact of a virtual educational intervention on medical students’ knowledge and attitudes towards patients with intellectual and developmental disabilities. J Intell Dev Disabil. 2023;48(1):91–9. 10.3109/13668250.2022.2112511 39815862

[17] Iacono T, Bigby C, Unsworth C, Douglas J, Fitzpatrick P. A systematic review of hospital experiences of people with intellectual disability. BMC Health Serv Res. 2014;14(1):1–8. 25344333 10.1186/s12913-014-0505-5 PMC4210514

[18] Boyle F, Dean J, Tran N, Lohan A, Thomas N, Biswas T, Salom C. Scoping and gap analysis of undergraduate resources in intellectual disability health.

[19] Trollor JN, Eagleson C, Ruffell B, Tracy J, Torr JJ, Durvasula S, Iacono T, Cvejic RC, Lennox N. Has teaching about intellectual disability healthcare in Australian medical schools improved? A 20-year comparison of curricula audits. BMC Med Educ. 2020;20:1–0. 10.1186/s12909-020-02235-w PMC7507627 32958040

[20] Trollor JN, Ruffell B, Tracy J, Torr JJ, Durvasula S, Iacono T, Eagleson C, Lennox N. Intellectual disability health content within medical curriculum: an audit of what our future doctors are taught. BMC Med Educ. 2016;16:1–9. 27066776 10.1186/s12909-016-0625-1 PMC4827238

[21] Moyle JL, Iacono T, Liddell M. Knowledge and perceptions of newly graduated medical practitioners in Malaysia of their role in medical care of people with developmental disabilities. J Policy Pract Intellect Disabil. 2010;7(2):85–95.

[22] Burge P, Ouellette-Kuntz H, Isaacs B, Lunsky Y. Medical students’ views on training in intellectual disabilities. Can Fam Physician. 2008;54(4):568–71. 18411386 PMC2294093

[23] Jiwa MI, Coret A, Sawyer A, Lunsky Y. Developmental disabilities in undergraduate medical education: The university of toronto experience. J Dev Disabil. 2020;25(2):1–4.

[24] Janicki MP, World Health Organization. Ageing and intellectual disabilities: improving longevity and promoting healthy ageing-summative report.

[25] Sullivan WF, Heng J, Cameron D, Lunsky Y, Cheetham T, Hennen B, Bradley EA, Berg JM, Korossy M, Forster-Gibson C, Gitta M. Consensus guidelines for primary health care of adults with developmental disabilities. Can Fam Physician. 2006;52(11):1410–8. 17279198 PMC1783706

[26] Burge P, Ouellette-Kuntz H, McCreary B, Bradley E, Leichner P. Senior residents in psychiatry: Views on training in developmental disabilities. Can J Psychiatry. 2002;47(6):568–71. 12211886 10.1177/070674370204700610

[27] Lennox N, Diggens J. Medical education and intellectual disability: A survey of Australian medical schools. J Intellect Dev Disabil. 1999;24(4):333–40.

[28] Werner S, Stawski M. Mental health: Knowledge, attitudes and training of professionals on dual diagnosis of intellectual disability and psychiatric disorder. J Intellect Disabil Res. 2012;56(3):291–304. 21554470 10.1111/j.1365-2788.2011.01429.x

[29] Morin D, Valois P, Crocker AG, Robitaille C, Lopes T. Attitudes of health care professionals toward people with intellectual disability: a comparison with the general population. J Intellect Disabil Res. 2018;62(9):746–58. 29968307 10.1111/jir.12510

[30] Mason J, Scior K. Diagnostic overshadowing’amongst clinicians working with people with intellectual disabilities in the UK. J Appl Res Intellect Disabil. 2004;17(2):85–90.

[31] Minihan PM, Bradshaw YS, Long LM, Altman W, Perduta-Fulginiti S, Ector J, Foran KL, Johnson L, Kahn P, Sneirson R. Teaching about disability: Involving patients with disabilities as medical educators. Disabil Stud Q. 2004;24(4).

[32] Hall I, Hollins S. Changing medical students’ attitudes to learning disability. Psychiatric Bulletin-Royal Coll Psychiatrists. 1996;20:429–30.

[33] Tracy J, Graves P. Medical students and people with disabilities: A teaching unit tor medical students exploring the impact of disability on the individual and the family. Med Teach. 1996;18(2):119–24.

[34] Tracy J, Iacono T. People with developmental disabilities teaching medical students–does it make a difference? J Intellect Dev Disabil. 2008;33(4):345–8. 19039695 10.1080/13668250802478633

[35] Boyd K, Bridge E, Mcconnell M, Kates N, Stobbe K. A curriculum of caring for people with developmental disabilities in medical education. J Dev Disabil. 2019;24(2):10–8.

[36] Tarzi G, Thakur A, Bobbette N, Pilatzke M, Lefkowitz G, Thomson K, Thatcher A, Hasan S, Fogle A, Blake M, Hines A. Evaluation of an Interprofessional Educational Intervention in Mental Health and Intellectual and Developmental Disability for Health and Social Service Trainees. Acad Psychiatry. 2024;20:1–6. 10.1007/s40596-024-02063-w PMC11634927 39567470

